# Wogonin inhibits multiple myeloma-stimulated angiogenesis via c-Myc/VHL/HIF-1α signaling axis

**DOI:** 10.18632/oncotarget.6796

**Published:** 2015-12-30

**Authors:** Rong Fu, Yan Chen, Xiao-Ping Wang, Teng An, Lei Tao, Yu-Xin Zhou, Yu-Jie Huang, Bao-An Chen, Zhi-Yu Li, Qi-Dong You, Qing-Long Guo, Zhao-Qiu Wu

**Affiliations:** ^1^ Department of Physiology, State Key Laboratory of Natural Medicines and Jiangsu Key Laboratory of Carcinogenesis and Intervention, China Pharmaceutical University, Nanjing 210009, China; ^2^ Department of Hematology and Oncology, Zhongda Hospital, Medical School, Southeast University, Nanjing 211189, China; ^3^ Department of Medicinal Chemistry, China Pharmaceutical University, Nanjing 210009, China; ^4^ Jiangsu Key Laboratory of Drug Design and Optimization, China Pharmaceutical University, Nanjing 210009, China

**Keywords:** wogonin, angiogenesis, VHL, c-Myc, multiple myeloma

## Abstract

Angiogenesis is associated with the progression of multiple myeloma (MM). Wogonin is an active mono-flavonoid with remarkable antitumor activity. However, its impact on MM-stimulated angiogenesis remains largely unknown. Here, we demonstrated that wogonin decreased expression and secretion of pro-angiogenic factors in MM cells via c-Myc/HIF-1α signaling axis, reducing MM-stimulated angiogenesis and MM cell proliferation *in vivo*. Overexpression of c-Myc in MM cells disrupted the balance between VHL SUMOylation and ubiquitination, and thus inhibited proteasome-mediated HIF-1α degradation. Impaired function of VHL ubiquitination complex in c-Myc-overexpressing cells was fully reversed by wogonin treatment via increasing HIF-1α-VHL interaction and promoting HIF-1α degradation. Collectively, our *in vitro* and *in vivo* studies reveal for the first time that wogonin represses MM-stimulated angiogenesis and tumor progression via c-Myc/VHL/HIF-1α signaling axis.

## INTRODUCTION

Multiple myeloma (MM) is an incurable monoclonal B-malignancy, characterized by the accumulation of malignant plasma cells within the bone marrow (BM). It has been well-documented that angiogenesis in the BM plays a critical role in supporting the growth, survival, progression, and drug resistance acquisition of the malignant clone. A growing body of evidence indicates that increased BM vascularization is associated with advanced MM stage and poor prognosis [1–3]. Pro-angiogenic factors (eg. VEGF, PDGF and bFGF) secreted by MM cells play a crucial role in controlling BM angiogenesis and the pathogenesis of a wide range of MM types [4, 5].

HIF-1α, the key mediator of the hypoxic response in cellular tissues, is a potent pro-angiogenic transcription factor, predominantly via induction of pro-angiogenic factor transcription [6]. Under normoxia, HIF-1α is hydroxylated by prolyl hydroxylase (PHD) and then binds to VHL for proteasome-mediated degradation through formation of the EC2V (Elongin BC-CUL2-VHL) E3 ubiquitin ligase complex. In a hypoxic environment, this hydroxylation-mediated degradation pathway is blocked, which results in HIF-1α translocation and accumulation in the nucleus, where it transactivates hypoxia-responsive genes, including VEGF that are implicated in endothelial cell invasion and angiogenesis [7, 8]. Clinical studies have indicated that MM cells often exhibit increased rates of angiogenesis, even in the presence of adequate oxygen concentrations [8-10]. At least 40% of MM presenting with significant amplification of c-Myc and overexpression of c-Myc likely occurs in MM through other mechanisms [3, 11]. Specifically, the c-Myc/HIF-1α signaling axis modulates MM cell production of pro-angiogenic factors and regulates angiogenesis under both normoxia and hypoxia [3]. Based on these findings, c-Myc/HIF-1α signaling axis appears to be a potential target for novel anti-angiogenesis agents in treatment of MM.

Although increased rates of angiogenesis normally occur under hypoxic conditions, it has been noted recently that c-Myc may prevent HIF-1α degradation via the dysregulation of VHL function [3, 8]. This finding, therefore, could not exactly explain the weakening of the HIF-1α-VHL interaction in conditions of c-Myc overexpression, and raises the possibility that modification of VHL may contribute to the dysregulated interaction. The main function of VHL is viewed as an adapter for many binding partners to form a functional E3 ligase complex to target HIF-1α degradation. Many reports have suggested that SUMOylated VHL by PIASy (a SUMO E3 ligase) localizes to the nucleus, while ubiquitylated VHL is cytoplasmic, and disables its function related to the inhibition of HIF-1α expression and angiogenesis [12, 13].

**Figure 1:**
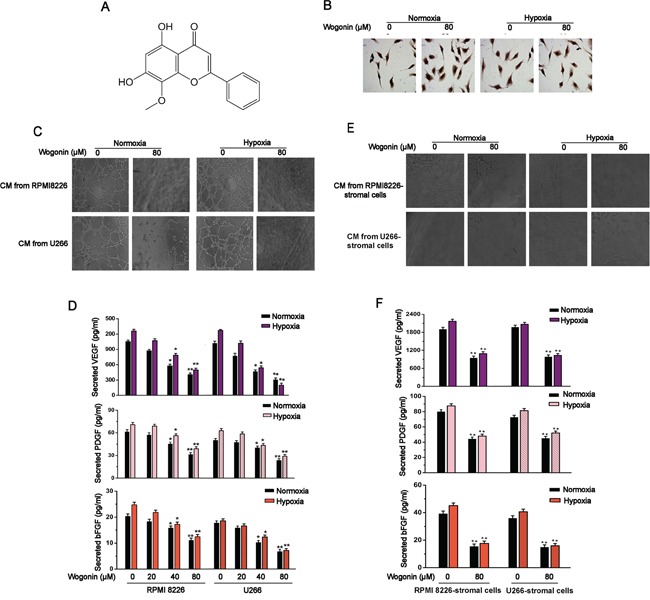
Inhibitory Effects of Wogonin on MM-Stimulated Angiogenesis **A.** Chemical structure of wogonin. **B.** MM cells were treated with wogonin at 80μM for 24 h under normoxia and hypoxia. HUVECs were cultured in conditioned medium from indicated MM cells for 8h and stained with Ki67. **C** and **D.** Effect of wogonin on MM-stimulated angiogenesis and secretion levels of pro-angiogenic factors. MM cells were treated as described in (B), and conditioned medium was collected for tube formation assays performed in HUVECs (C). MM cells were treated with various concentrations of wogonin under normoxia and hypoxia, and ELISA was performed to measure secreted VEGF, PDGF and bFGF levels in the collected conditioned medium (D). **E** and **F.** Effect of wogonin on MM/stromal cells-stimulated angiogenesis and secretion levels of pro-angiogenic factors. MM cells co-cultured with stromal cells were treated as described in *B*, and conditioned medium was collected for tube formation assays performed in HUVECs (E). MM cells were treated as described in (D), and ELISA was performed to measure secreted VEGF, PDGF and bFGF levels in the collected conditioned medium (F). Magnification of representative images (C and E) is 200×. Data are shown as means ± SEM (n = 3). *p < 0.05, **p < 0.01, one-way ANOVA (D) or two-paired Student's t-test (F).

Wogonin, one of the active mono-flavones in the most popular Chinese herbal remedy Huang-Qin (*Scutellaria baicalensis* Georgi) (Fig. 1A), has been recognized as an anticancer drug candidate with potentially low toxicity [14]. Recently, the anti-angiogenic activity of wogonin has been reported in MCF-7 breast cancer cells [15]. However, the underlying mechanisms remain largely unclear. Here, we have determined the effect of wogonin on MM-induced angiogenesis *in vitro* and *in vivo*, using the established MM cell lines as well as patient-derived-MM cells. Moreover, we have discovered for the first time that wogonin inhibits MM-induced angiogenesis as well as MM cell proliferation *in vivo* via c-Myc/VHL/HIF-1α signaling axis.

## RESULTS

### Wogonin inhibited MM-stimulated angiogenesis via decreasing secretion levels of pro-anigogenic factors

MM cells treated with wogonin at doses of 20, 40 or 80 μM did not display a defect in proliferation, as monitored by MTT assay and Ki67 immunohistochemistry staining (Fig. 1B; Fig. S1A and S1B). We then determined whether wogonin at the non-cytotoxic dose could affect MM-induced angiogenesis *in vitro*. Results from wound healing and transwell migration assays showed that migration of human umbilical endothelial cells (HUVECs) was strongly repressed when cultured in the presence of conditioned medium derived from wogonin-treated MM cells under both normoxic and hypoxic culture conditions (Fig. S1C, S1D and S1E). To further determine whether wogonin could inhibit MM-induced angiogenesis, we performed a tube formation assay *in vitro*. As shown in Fig. 1C, HUVECs cultured in conditioned medium from wogonin-treated MM cells almost completely lost their ability to form tubular structures as compared to cells cultured in conditioned medium derived from untreated cells. Taken together, our results suggested that wogonin even at non-cytotoxic dose could inhibit MM-induced EC migration and tubular formation *in vitro*. Angiogenesis in bone marrow largely depends on MM-secreted pro-angiogenic factors (*e.g.* VEGF, PDGF and bFGF). We therefore sought to test whether wogonin treatment could affect secretion of these pro-anigogenic factors in MM cells. For this purpose, MM cells (RPMI 8226 and U266) were cultured in the absence or presence of wogonin under both normoxic and hypoxic conditions for 24 h, and the secretion levels of VEGF, PDGF and bFGF in culture medium were detected by ELISA. Basal levels of secreted VEGF, PDGF and bFGF were slightly increased under hypoxic condition as compared to normoxic condition (Fig. 1D). Wogonin treatment decreased secretion levels of these pro-angiogenic factors in both cell lines under both culture conditions in a dose-dependent manner (Fig. 1D).

### Wogonin inhibited angiogenesis in MM-stromal cell co-cultures

It is well conceived that MM cells expand in the BM microenvironment and induce angiogensis through cross-talk with stroma cells and extracellular matrix [16]. To investigate the anti-angiogenic effect of wogonin, we sought to establish an *in vitro* co-culture system that could recapitulate the BM microenvironment *in vivo*. MM cells co-cultured with stromal cells were treated with wogonin for 24h, and the conditioned medium was collected for angiogenesis assays. As shown in Fig. 1E, tube forming potentials of HUVECs were markedly repressed when cultured with conditioned medium from wogonin-treated MM-stromal cells. Interestingly, dramatically higher secretion levels of the pro-angiogenic factors were detected in conditioned medium from MM-stromal cells than those from MM cells alone (Fig. 1F). Furthermore, secretion levels of the pro-angiogenic factors were significantly repressed in wogonin-treated MM-stromal cells as compared to control cells (Fig. 1F).

### Wogonin inhibited c-Myc expression and promoted HIF-1α degradation in MM cells

**Figure 2:**
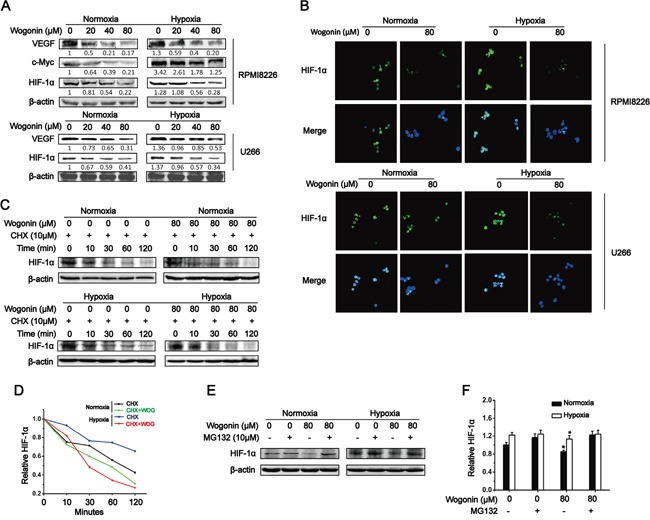
Wogonin Inhibited c-Myc Expression and Promoted HIF-1α Degradation in MM cells **A.** RPMI 8226 cells (top) or U266 cells (bottom) were treated with various concentrations of wogonin (0, 20, 40 and 80μM) for 24 h under normoxia and hypoxia. Cell lysates were prepared and subjected to Western blot analysis using the indicated antibodies. **B.** RPMI 8226 and U266 cells were treated with wogonin (80μM) for 24h under normoxia and hypoxia, and subjected to HIF-1α immunofluorescent staining. Nuclei are counter stained with DAPI (blue). Magnification, 400×. **C.** RPMI 8226 cells were treated with cycloheximide (CHX) for 0, 10, 30, 60 and 120min under normoxia and hypoxia in the presence or absence of wogonin (80μM). Cell lysates were prepared and subjected to immunoblotting analysis using the indicated antibodies. **D.** Quantification of HIF-1α protein levels shown in (C). **E.** RPMI 8226 cells were treated with the proteasome inhibitor, MG132 for 6h under normoxia and hypoxia with or without wogonin. Cell lysates were prepared and subjected to immunoblotting analysis using an anti-HIF-1α antibody. **F.** Quantification of HIF-1α protein levels shown in (E). Data are shown as means ± SEM (n = 3). *p < 0.05, one-way ANOVA.

We next sought to determine whether wogonin treatment could affect expression levels of VEGF in MM cells. Results from Western blot and RT-qPCR showed that VEGF expression at both protein and mRNA levels were markedly decreased in wogonin-treated MM cells as compared to those in control cells (Fig. 2A; Fig. S2A, *left panel*). As c-Myc/HIF-1α signaling axis is reported to play a critical role in angiogenesis via controlling VEGF expression and secretion, we sought to examine whether wogonin could affect c-Myc/HIF-1α signaling axis in MM cells. We have found that wogonin dose-dependently reduced expression of c-Myc and HIF-1α at protein but not mRNA level in RPMI 8226 and U266 cells under both normoxic and hypoxic conditions (Fig. 2A; Fig. S2A, *right two panels*). Immunofluorescence analysis further showed that wogonin strongly decreased expression of HIF-1α in nucleus in both cell lines (Fig. 2B). Interestingly, we found that expression level of c-Myc were ∼4.5 folds higher in RPMI 8226 cells than in U266, consistent with the previous report (Fig. S2B and S2C) [8]. We thus chose RPMI 8226 cells for further study.

As wogonin suppressed HIF-1α protein expression without affecting its mRNA level, we proposed that wogonin down-regulated HIF-1α protein level through promoting its degradation. To test this, we used cycloheximide (CHX) to block new protein synthesis and then assessed HIF-1α protein level at 120 min post CHX treatment in RPMI 8226 cells under both normoxic and hypoxic conditions. Consistent with previous report [8], we found that HIF-1α had an extremely long half-life (∼90 min and >120 min under normoxia and hypoxia, respectively). HIF-1α half-life in wogonin-treated cells was reduced to ∼60 min and ∼30 min under normoxia and hypoxia, respectively (Fig. 2C; quantification shown in Fig. 2D). These results indicated that wogonin repressed HIF-1α protein level through promoting its degradation. To further confirm the results, we used the proteasomal inhibitor, MG132, to block HIF-1α degradation and assessed HIF-1α protein levels under both culture conditions. As expected, MG132 just slightly increased HIF-1α protein level in untreated cells as HIF-1α protein in the cells is extremely stable (compare lane 2 versus 1 in Fig. 2E; quantification shown in Fig. 2F). In striking contrast, wogonin-treated cells markedly decreased HIF-1α protein level and thus were more sensitive to MG132 treatment especially when cultured under normoxic condition (compare lane 4 versus 3 in Fig. 2E; Fig. 2F). Taken together, these results suggested that wogonin promoted HIF-1α degradation in MM cells under both normoxia and hypoxia.

### Wogonin inhibited c-Myc-mediated HIF-1α accumulation

**Figure 3:**
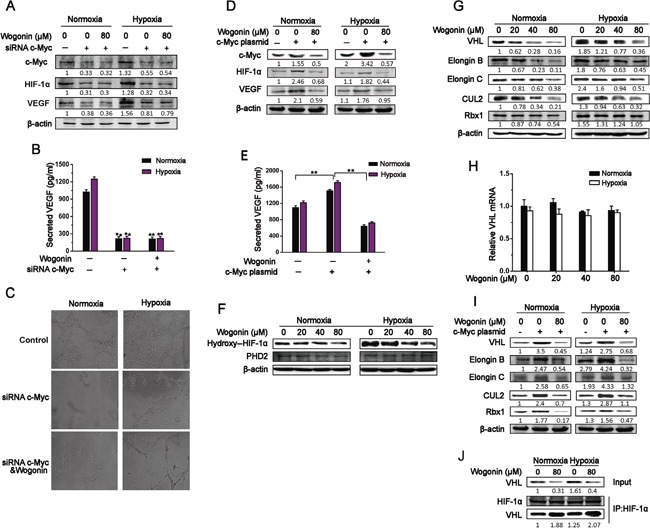
Wogonin Reduced c-Myc-Mediated Accumulation of VHL Complex **A** and **B.** RPMI 8226 cells transfected with control or c-Myc siRNA for 24h were cultured with or without wogonin (80μM) under normoxia and hypoxia for another 24h. Cell lysates (A) and conditioned medium (B) were collected and subjected to Western blotting and ELISA assays, respectively. Data are shown as means ± SEM (n = 3). **p < 0.01, Student's t-test. **C.** HUVECs were cultured atop of Matrigel in conditioned medium as described in (A), and tube formation was evaluated by phase contrast microscopy (magnification, 200×) 8h later. **D.** RPMI 8226 cells transfected with mock or c-Myc expressing vectors were cultured under normoxia and hypoxia for 24h. Cells were treated with wogonin (0 and 80μM) for an additional 24h and harvested for immunoblot analysis with the indicated antibodies. **E.** Cells were treated as in *D*, and VEGF levels in conditioned medium were mesured by ELISA. Data are shown as means ± SEM (n = 3). **p < 0.01. **F** and **G.** Cells were treated with various concentrations of wogonin (0, 20, 40 and 80μM) for 24h under normoxia and hypoxia. Cell lysates were prepared and subjected to Western blot analysis using the indicated antibodies to PHD2 (F) and VHL complex components (G). **H.** Cells were treated as in described in (F), and cell lysates were prepared and subjected to RT-qPCR analysis. **I.** RPMI 8226 cells were transfected with control or c-Myc plasmids and cultured under normoxia and hypoxia for 24 h. Cells were then treated with wogonin for another 24h, and cell lysates were prepared and subjected to Western blot analysis using antibodies as described in (G). **J.** RPMI 8226 cells were cultured under normoxia and hypoxia in the presence or absence of wogonin for 24h. Cell lysates were prepared and subjected to anti-HIF-1α immunoprecipitation (IP) assay, followed by immunoblotting analysis using antibodies to VHL and HIF-1α. Data are shown as means ± SEM (n = 3). **p < 0.01, one-way ANOVA.

To further determine if wogonin repressed protein levels of HIF-1α and VEGF in a c-Myc-dependent manner, we used RNA interfere (RNAi) to deplete endogenous c-Myc in RPMI 8226 cells. As shown in Fig. 3A, c-Myc protein was efficiently depleted in c-Myc siRNA-transfected cells. Meanwhile, c-Myc depleted-cells with reduced expression levels of HIF-1α and VEGF displayed a markedly impaired potential to form tubular structures when cultured atop of Matrigel (Fig. 3A-3C). Wogonin treatment in c-Myc silensced-cells did not further decrease expression levels of HIF-1α and VEGF or strengthen the impaired potential to form tubular structures. Next, we overexpressed c-Myc in RPMI 8226 cells and cultured the cells in the presence of wogonin. As expected, c-Myc over-expressing cells enhanced expression and secretion levels of HIF-1α and VEGF, which could be effectively abolished when cultured in the presence of wogonin (Fig. 3D and 3E). In consistent with these results, HUVECs cultured in conditioned medium from c-Myc overexpressing cells displayed a slight increase in migration potentials as compared to cells in conditioned medium from control vector expressing cells, and the increased migration potentials could be sufficiently abolished by wogonin treatment (Fig. S3).

### Wogonin reduced c-Myc-mediated accumulation of VHL complex

As HIF-1α degradation is primarily controlled by proline hydroxylase domain protein 2 (PHD2) or von Hippel-Lindau (VHL) E3 ubiquitin ligase complex, we first assessed whether wogonin increased PHD2 expression, thus promoting HIF-1α degradation. Interestingly, wogonin did not affect PHD2 expression even at the concentration of 80 μM, but decreased expression level of hydroxyl-HIF-1α in a dose-dependent manner (Fig. 3F). We next sought to determine whether wogonin regulated expression of VHL complex components, thus affecting HIF-1α degradation. Unexpectedly, we found that wogonin-treated cells under normoxic and hypoxic conditions displayed a robust decrease in expression of all the VHL complex components (*e.g.* VHL, CUL2, Rbx1, Elongin B and Elongin C) (Fig. 3G). RT-qPCR analysis revealed that VHL mRNA level was comparable in control and wogonin-treated cells (Fig. 3H). We further tested whether wogonin affected expression of VHL complex components in c-Myc over-expressing cells. The c-Myc over-expressing cells markedly up-regulated expression levels of VHL complex components, and c-Myc-increased expression of VHL complex components was sufficiently abolished in wogonin-treated cells (Fig. 3I). As wogonin decreased HIF-1α expression in tandem with a robust reduction in VHL expression in MM cells, we hypothesized that wogonin may affect modification of VHL E3 ubiquitin ligase complex and thus impact the complex-mediated regulation of HIF-1α in the cells. It has been reported that c-Myc promotes VHL modification, affecting formation of the VHL complex and thus repressing its function [8]. To test our hypothesis, we performed an anti-HIF-1α immunoprecipitation assay to assess whether the association between VHL and HIF-1α was altered following wogonin treatment. Interestingly, we found that the interaction between HIF-1α and VHL was markedly increased in wogonin-treated cells under both normoxic and hypoxic conditions (Fig. 3J).

### Wogonin abolished c-Myc-mediated regulation of VHL SUMOylation and ubiquitination

**Figure 4:**
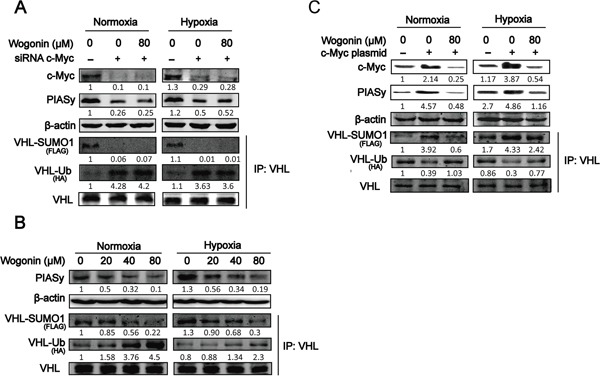
Wogonin Abolished c-Myc-Mediated Regulation of VHL SUMOylation and Ubiquitination **A.** RPMI 8226 cells were transfected with control siRNA (or c-Myc siRNA), FLAG-SUMO1 and HA-Ubiquitin plasmids for 24 h, followed by incubation with or without wogonin at 80μM under both conditions for another 24h. Cell lysates were prepared and subjected to Western blot and IP assays using the indicated antibodies. **B.** Cells were cultured under both conditions in the presence of various concentrations of wogonin for 24h. Cell lysates were prepared and subjected to Western blot and IP assays using the antibodies as described in (A). **C.** Cells transfected with mock or c-Myc expressing vector were cultured under both conditions for 24h. Cells were then treated with wogonin or vehicle for another 24h, and harvested for Western blot and IP analyses using the antibodies as described in (A).

SUMO1 modification of VHL increases protein stability and disables its inhibitory effect on HIF-1α transcriptional activity. PIASy, a SUMO E3 ligase, has been reported to stimulate VHL SUMOylation leading to a decrease in VHL ubiquitination. To further understand the underlying mechanism by which c-Myc regulates VHL modification, cells were co-transfected with c-Myc siRNA, FLAG-SUMO1 and HA-Ubi, and VHL modification was assessed. As shown in Fig. 4A, c-Myc siRNA transfected cells with markedly reduced expression of c-Myc displayed a robust decrease in PIASy expression. Co-immunoprecipitation (co-IP) assay further revealed a robust reduction in SUMOylated VHL in tandem with an increase in ubiquitinated VHL in VHL co-IP complex in c-Myc depleted cells (Fig. 4A). Wogonin treatment did not affect PIASy expression level or posttranscriptionally modified VHL in VHL co-IP complex in c-Myc depleted cells (Fig. 4A). To directly determine whether wogonin treatment could affect SUMOylation or ubiquitination of VHL, MM cells co-treanfected with FLAG-SUMO1 and HA-Ubi were treated with wogonin at different doses and subjected to co-IP analysis using an anti-VHL antibody. The levels of PIASy expression as well as SUMOylated VHL in VHL co-IP complex were dose-dependently reduced whereas ubiquitinated VHL in the complex was increased in a dose-dependent manner (Fig. 4B). To further determine whether wogonin treatment could abolish c-Myc-induced modifications of VHL, MM cells were co-transfected with c-Myc, FLAG-SUMO1 and HA-Ubi, cultured in the absence or presence of wogonin and subjected to anti-VHL co-IP analysis. As shown in Fig. 4C, c-Myc overexpressing cells displayed increased levels of SUMOylated VHL in VHL co-IP complex in tandem with a robust reduction in ubiquitinated VHL level in the co-IP complex, which could be fully reversed by wogonin treatment.

### Wogonin inhibited tumor angiogenesis and growth *in vivo*

**Figure 5:**
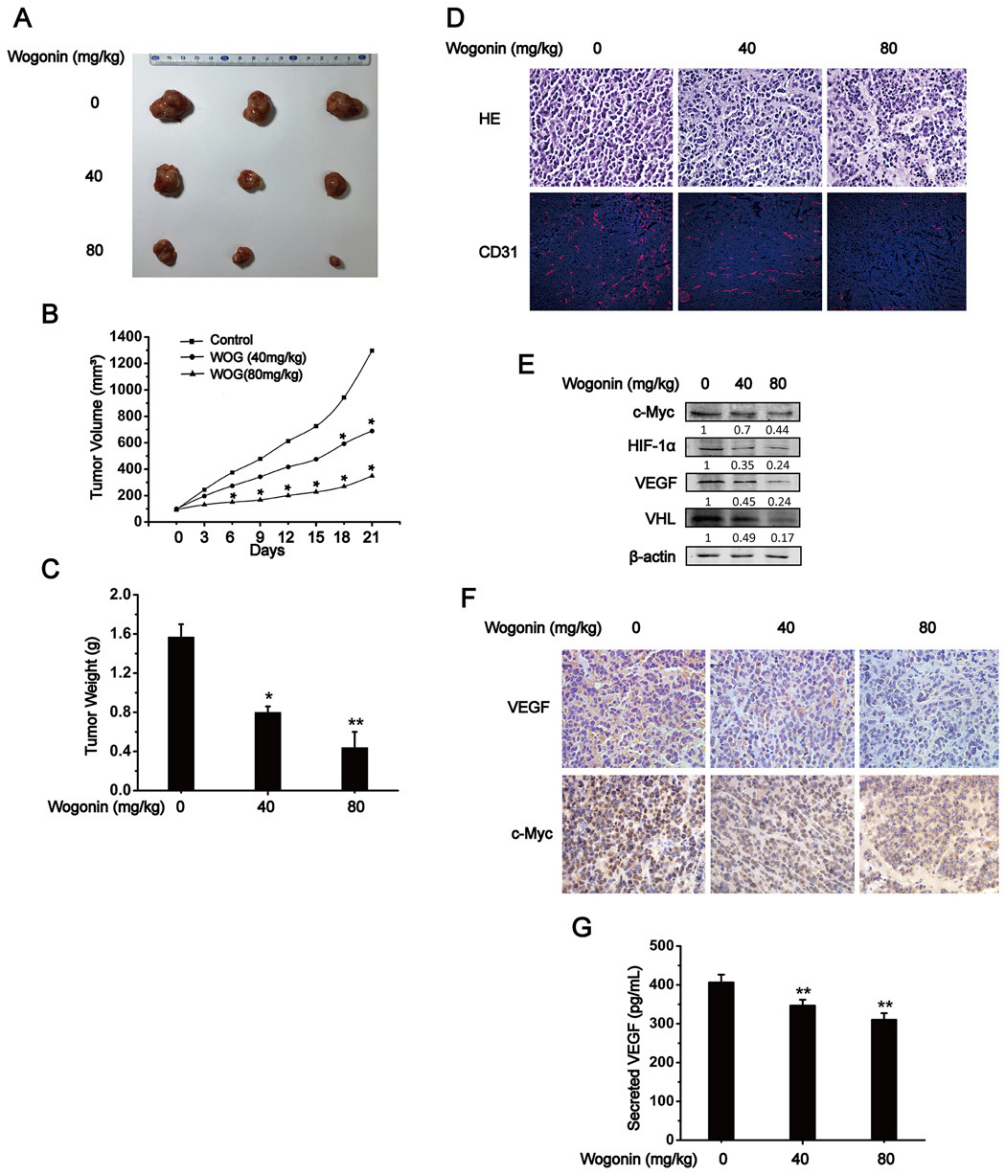
Wogonin Inhibited Tumor Angiogenesis and Growth *in vivo* **A, B** and **C.** 1×10^6^ RPMI 8226 cells were subcutaneously (*s.c.*) injected in nude mice and the tumor bearing mice were randomly divided into 3 groups. Wogonin was administrated at doses of 0, 40, 80mg/kg via intravenous (*i.v.*) injection every three days, and tumors were retrieved at 21 days post wogonin administration. Representative images of tumors from 3 groups were shown (A), and tumor volumes (B) as well as tumor weight (C) were measured. **D.** Tumors were retrieved and sectioned for histological analysis (H.E. staining, *top*) and anti-CD31 immunofluoscent analysis (*bottom*). **E.** Tumor lysates from three groups were prepared and subjected to Western blot analysis using the indicated antibodies. **F.** Immunohistochemistry analysis of c-Myc and VEGF (magnification, 400×). **G.** Secretion of VEGF in plasma derived from vehicle- and wogonin-treated mice was measured by ELISA. Data are shown as means ± SEM (n = 3). *p < 0.05, **p < 0.01, one-way ANOVA.

We next tested whether wogonin inhibited tumor angiogenesis and growth *in vivo*. For this purpose, 1×10^6^ RPMI 8226 cells were subcutaneously (*s.c.*) injected in nude mice and the tumor bearing mice were randomly divided into 3 groups. Wogonin was administrated at doses of 0, 40, 80 mg/kg via intravenous (*i.v.*) injection every three days. Administration of wogonin at 40 and 80 mg/kg markedly repressed tumor growth as indicated by gross examination, tumor volume and tumor weight (Fig. 5A, 5B and 5C). Tumor volume and weight were reduced by > 70% in 80 mg/kg wogonin-treated mice than in vehicle-treated mice (Fig. 5B and 5C). Immunofluorescence staining with an anti-CD31 antibody revealed that micro-vessel density (*i.e.* CD31^+^ cells per field) was strongly reduced within tumors derived from wogonin-treated mice (Fig. 5D). Cell lysates of the indicated tumors were prepared and subjected to Western blot analysis. As shown in Fig. 5E, expression levels of c-Myc, HIF-1α, VHL and VEGF were dramatically decreased in tumors from wogonin-treated mice, which is consistent with our observations *in vitro*. Immunohistochemistry staining further showed that wogonin repressed expression of VEGF and c-Myc in a dose-dependent manner, and few c-Myc or VEGF staining positive cells could be observed in tumors from 80 mg/kg wogonin-treated mice (Fig. 5F). Moreover, we observed markedly reduced VEGF secretion in peripheral blood of wogonin-treated mice (Fig. 5G). Importantly, we did not observe any defects in body weight or hematological parameters, or any histological changes in vital organs (*e.g.* heart, liver, lung, kidney and spleen) in wogonin-treated mice (Fig. S4A and S4B; Table S1), suggesting that wogonin when administrated systematically exhibited potent anti-tumor activity but did not affect physiological functions of vital organs.

### Wogonin synergistically repressed MM-stimulated angiogenesis with bortezomib or lenalidomide and inhibited expression of c-Myc and HIF-1α in patient-derived MM cells

**Figure 6:**
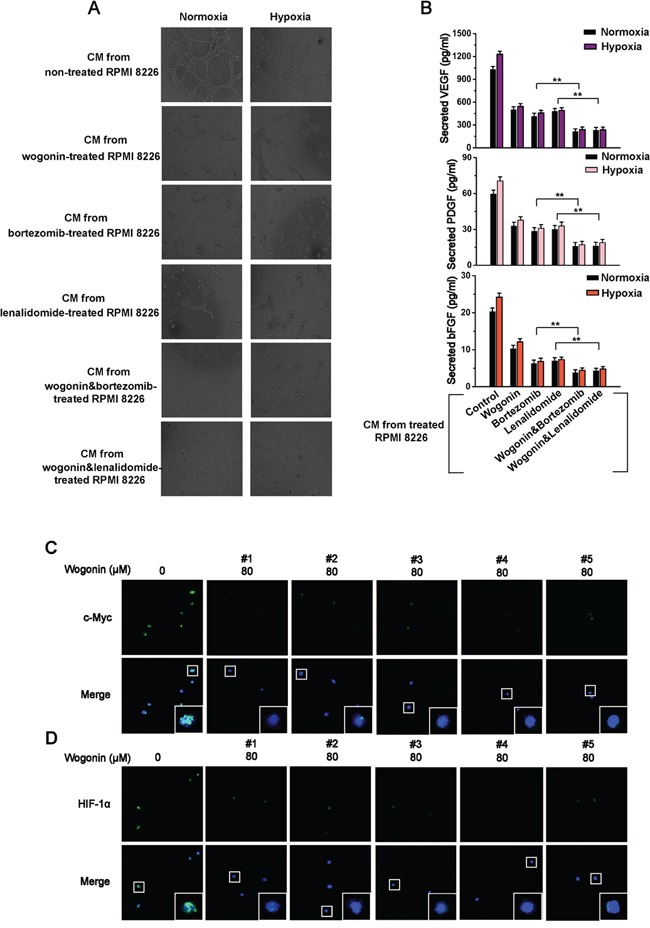
Wogonin Synergistically Repressed MM-Stimulated Angiogenesis with Bortezomib or Lenalidomide And Inhibited Expression of c-Myc and HIF-1α in Patient-Derived MM Cells **A.** RPMI 8226 were incubated with wogonin (40μM), bortezomib (10 nM), lenalidomide (10μM), wogonin (40μM) plus bortezomib (10 nM), or wogonin (40μM) plus lenalidomide (10μM) under normoxia and hypoxia for 24h. Conditioned medium was collected for tube formation assays performed in HUVECs. Tube formation was evaluated by phase contrast microscopy (magnification, 200×). **B.** MM cells were treated as described in (A), and ELISA was performed to measure secreted VEGF, PDGF and bFGF levels in the collected conditioned medium. Data are shown as means ± SEM (n = 3). **p < 0.01, Student's t-test. **C** and **D.** Primary MM cells were treated with wogonin (80μM) for 24h and subjected to confocal immunofluorescent analysis using antibodies to c-Myc (C) and HIF-1α (D). Nuclei are counter stained with DAPI (blue). Insets, higher magnification of boxed areas. Magnification, 200×. Data are shown as means ± SEM (n = 3). *p < 0.05, **p < 0.01, one-way ANOVA.

We sought to determine whether wogonin could exert a synergistic inhibition on MM-stimulated angiogenesis when combined with bortezomib or lenalidomide, two of the most-widely used agents for MM treatment. For this purpose, conditioned medium from MM cells that were treated with wogonin-, bortezomib-, lenalidomide-, or in combination were collected and subjected to HUVEC tube formation or ELISA assays. As shown in Fig. 6A, tube forming potentials of HUVECs were strongly repressed when cultured in conditioned medium from MM cells that were treated with wogonin, bortezomib or lenalidomide alone, with a further repression observed in the combination. Similarly, wogonin synergistically inhibited secretion levels of pro-angiogenic factors (e.g. VEGF, PDGF and bFGF) in MM cells with bortezomib or lenalidomide (Fig. 6B). Collectively, these results suggested wogonin synergistically repressed MM-stimulated angiogenesis with bortezomib or lenalidomide. Finally, we sought to determine whether wogonin could inhibit expression of c-Myc and HIF-1α in patient-derived MM cells. To this end, MM cells from 5 patients without prior therapy were collected, treated with wogonin (80μM) and then subjected to immunofluorescence staining. We found that wogonin-treated MM cells displayed a striking reduction in expression of c-Myc and HIF-1α in the nucleus (Fig. 6C and 6D).

## DISCUSSION

As MM-stimulated angiogenesis is an essential process for MM cell proliferation and metastasis, strategies to inhibit angiogenesis have been investigated as novel anti-MM treatments [17, 18]. It has been reported that c-Myc/HIF-1α axis is crucial to induce expression and secretion of various pro-angiogenic factors (e.g. VEGF, PDGF and bFGF) [3]. Recent study has pointed out that activation of c-Myc/HIF-1α axis in MM cells independent of oxygen [3]. In the present study, we discovered that wogonin strongly inhibited MM-stimulated angiogenesis *in vivo* and *in vitro* via c-Myc/HIF-1α/VEGF signaling axis, suggesting that these findings may provide a rationale for wogonin's application in the treatment of MM.

The effect of wogonin on cell proliferation indicates that the viability of MM cells declines sharply by 50% at 143.2 μM wogonin. However, migratory and tube forming potentials of HUVECs displayed severely defective when cultured in conditioned medium from MM cells or MM-stromal cells that were treated with non-cytotoxic doses of wogonin. As VEGF, PDGF and bFGF are the main secretory cytokines in MM-stimulated angiogenesis, we applied ELISA to detect their secretion level in MM cells. As speculated, wogonin indeed decreased secretion levels of VEGF, PDGF and bFGF in MM cells. It has been well documented that c-Myc/HIF-1α axis plays a critical role in MM-stimulated angiogenesis in both normoxic and hypoxic tissues. It remains unclear whether wogonin affects MM-stimulated angiogenesis via c-Myc/HIF-1α/VEGF axis. Thus, the impact of wogonin on expression levels of c-Myc, HIF-1α and VEGF as well as VEGF secretion level in MM cells was determined. Our studies showed that wogonin inhibited expression levels of c-Myc and HIF-1α, causing a robust decrease in expression of VEGF at both protein and mRNA levels as well as secreted VEGF level. Moreover, wogonin treatment almost completely reversed up-regulated expression of HIF-1α and VEGF in Myc overexpressing MM cells, suggesting that wogonin inhibited MM-stimulated angiogenesis via c-Myc/HIF-1α/VEGF axis.

Most primary MM cells derived from patients with poor prognosis, even when cultured under normoxic conditions, display an robustly elevated expression level of c-Myc and HIF-1α [19-21]. We discovered that depletion of c-Myc in MM cells reduced HIF-1α and VEGF expression as well as MM-stimulated angiogenesis, whereas wogonin lost its anti-angiogenic effect in c-Myc-depleted MM cells. Therefore, we sought to identify key molecules that are responsible for wogonin-mediated regulation of c-Myc/HIF-1α/VEGF axis in wild-type and c-Myc overexpressing MM cells. Our results revealed that wogonin promoted HIF-1α degradation through proteasome/ubiquitination pathway, without affecting HIF-1α mRNA expression level. Of note, although PHD2 plays a key role in HIF-1α degradation [12], wogonin treatment had no effect on PHD2 protein expression. Previous data show that disruption of binding to the VHL complex and post-translational modifications of VHL are both involved in c-Myc-mediated HIF-1α stabilization [8]. Our observations revealed that wogonin treatment induced a marked decrease in expression levels of VHL complex in wild-type and c-Myc overexpressing MM cells. Interestingly, we observed that wogonin enhances the interaction between HIF-1α and VHL in MM cells, which likely contributed to the reduced accumulation of HIF-1α protein.

The underlying mechanism by which wogonin normalizes c-Myc-mediated inactivation of VHL function remains largely unknown. It has been reported that SUMO E3 ligase, PIASy, interacts with VHL and induces VHL SUMOylation on lysine residue 171, and dually-ubiquitinated lysine residues 171 and 196 can be removed by PIASy. Ubiquitilated VHL is localized predominantly in the cytoplasm, while SUMOylated VHL results in increased VHL protein stability and function [13]. It has been reported that c-Myc promotes VHL accumulation, abrogating its inhibitory effect on transcriptional activity of HIF-1α. In this study, we for the first time provided an evidence showing that c-Myc increased accumulation and stability of VHL protein via promoting VHL SUMOylation and thus repressing its ubiquitination. We further discovered that increased level of VHL SUMOylation and reduced level of VHL ubiquitination in c-Myc overexpressing MM cells were completely reversed by wogonin treatment, leading to a robust decease in VHL expression and protein function in wogonin-treated cells.

Based on the following observations that: 1) wogonin alone or in combination with bortezomib or lenalidomide strongly repressed MM-stimulated angiogenesis *in vitro* and *in vivo*, 2) wogonin markedly inhibited MM cell proliferation *in vivo*, and 3) wogonin dramatically inhibited expression levels of c-Myc and HIF-1α in advanced patient-derived MM cells, we propose that wogonin could be developed into a novel therapeutic agent for treatment of advanced MM.

## MATERIALS AND METHODS

### Reagents and mice

Wogonin was isolated from *S. baicalensis* Georgi according to the protocols reported previously with slight modifications [21]. Wogonin was dissolved in dimethylsulfoxide (DMSO) as a stock solution and stored at −20°C. Further dilution was performed in culture medium prior to each experiment (DMSO%<0.1%). 3-(4,5-dimethylthiazol-2-yl)-2,5-diphenyltetrazolium bromide (MTT) was obtained from Fluka and dissolved in 0.01 M PBS. The enzyme-linked immunosorbent assay (ELISA) kit for VEGF was purchased from BosterBio (Pleasanton, USA). The rabbit polyclonal antibodies to c-Myc, HIF-1α, VEGF, VHL, CUL2, Flag-tag and PIASy were purchased from Bioworld. The mouse polyclonal antibody to β-actin was obtained from Boster. Other antibodies to Rbx1, Elongin C, Elongin B and HA were from Santa Cruz Biotechnology. IRDyeTM 800 conjugated secondary antibodies were obtained from Rockland Inc. Five-to-six-week-old female BALB/c-nude mice (SLACAS, Shanghai, China) were used for xenograft assays. The mice were fed *ad libitum* throughout the experimental period.

### Cell culture

The human MM cell lines RPMI 8226 and U266 were purchased from American Type Culture Collection (ATCC, Manassas, VA, USA) and cultured in RPMI 1640 medium supplemented with 10% fetal bovine serum (Invitrogen). HUVECs were isolated from human umbilical cord veins by collagenase treatment as described previously [23]. The cells were grown in medium 199 (Invitrogen) containing endothelial cell growth supplement (ECGS, 30μg/ml, Sigma) and epidermal growth factor (EGF, 10 ng/ml, Sigma) supplemented with 10% fetal bovine serum. For normoxic and hypoxic culture, cells were grown in a humidified atmosphere with 21% and 1% oxygen supplies, respectively.

### Preparation of conditioned medium (CM)

MM cells were treated with wogonin, bortezomib and lenalidomide for 24 h, or transfected with the pcDNA3.1-c-Myc plasmid followed by treatment with wogonin (0 and 80μM) for 24 h. After wogonin treatment, the cells were washed three times in phosphate-buffered saline and fed with serum-free medium, and then the CM was collected 12 h later.

### *In vitro* MM-stromal cell co-cultures

Stromal cells were isolated from C57B6 mice at 6-8 weeks old as previously described [24]. The isolated stromal cells were cultured with DMEM medium supplemented with 10% FBS in 24-well plate. After obtaining a confluent feeder layer, the medium was changed to RPMI1640 medium supplemented with 10% FBS. MM cells were seeded atop of the stromal cells, and the conditioned medium from the co-cultures was collected for further use.

### MTT assay

MTT assay was performed according to the manufacturer's instruction. Briefly, 1×10^4^ cells were seeded in a 96-well plate and treated with wogonin at indicated concentrations for 24h. Twenty-μl MTT solution (5mg/ml in PBS) was added to each well and incubated for additional 3 h. The supernatants were removed and 100 μl DMSO was added and incubated with agitation for 5 min to ensure total solubility of formazan crystals. The optical density was measured at 570 nm.

### Scratch-wound assay

HUVECs were seeded in a 6-well plate and allowed to grow to 80% confluence. The cell monolayer was subsequently scratched with a pipette tip (Axygen) to create a narrow wound-like gap. Shortly after wounding, the cells were washed twice with PBS and incubated with VEGF (10 ng/ml) or CM of MM cell lines. The plates were photographed at 0 and 12h using an inverted light microscope. The number of migrated cells was quantified by manual counting and six randomly chosen fields were analyzed for each well.

### Cell migration assay

Cell migration was assessed using a modified Boyden chamber assay, as described previously [25]. Briefly, VEGF (10 ng/ml) or CM of MM cell lines was added to the lower chamber, and HUVECs were cultured in DMEM medium in the upper chamber. Following 4h culture, the membrane between the two chambers was fixed and stained. Cells that migrated and attached onto the lower surface of the membranes were counted. Five randomly chosen fields were counted for each group.

### Tube formation assay

The tube formation assay was performed on HUVECs to assess the ability of cells to form a 3-dimensionally organized capillary network. In brief, 4.5×10^4^ cells were seeded atop Matrigel (BD) layer in 96-well plate and cultured in CM or regular medium supplemented with VEGF (10 ng/ml). Following culture for 8 h, the tubular structures were examined using a phase-contrast photomicroscope, with planimetric parameters measured by computed image analysis.

### Measurement of pro-angiogenic factor secretion level

Levels of secreted VEGF, PDGF and bFGF in MM cells were measured using a sandwich ELISA kit according to the manufacturer's protocol.

### RT-qPCR analysis

Total RNA was extracted, reversely transcribed and subjected to qPCR analysis. All qPCR data were analyzed using the 2^−ΔΔCT^ method and expressed relative to the untreated sample. The sequences of indicated primers were listed in Table S2.

### Cell transfection

For gene knockdown experiments, c-Myc siRNA and non-targeting scrambled siRNA (NS) obtained from Santa Cruz Biotech were transfected into RPMI 8226 cells using PepMute™ siRNA transfection reagent (SignaGen Laboratories). For gene overexpression experiments, cells were transfected with pcDNA3.1-c-Myc, Flag-SUMO1 or HA-Ubiquitin plasmids (Addgene) using GenJet™ *in vitro* DNA transfection reagent (SignaGen Laboratories).

### Immunoblotting and immunoprecipitation

Western blotting was carried out as previously reported [26]. Proteins were separated on 8%, 10% or 12% Tris-Glycine SDS-PAGE and transferred to nitrocellulose membranes (BioTrace). Detection was performed using the Odyssey Infrared Imaging System (LI-COR Inc). Immunoprecipitation was conducted as follows: 500 μg cell lysates were incubated with 1 μg primary antibody at 4°C for 1 h, followed by incubation with 20 μl protein A/G PLUS agarose at 4°C overnight. The beads were collected and washed with ice-cold lysis buffer and subjected to SDS-PAGE.

### Immuno-cytochemical and -histochemical staining

Immunocytochemical staining was performed as described previously with some modifications [27]. Briefly, cells seeded on glass slides were fixed with 4% paraformaldehyde (PFA), washed with ice-cold PBS and permeabilized with Triton X-100 in PBS. Slides were blocked with 5% BSA for 1h and incubated with anitbodies against HIF-1α Ab or c-Myc Ab at 4°C overnight, followed by incubation with donkey anti-rabbit IgG-Alexa 488 secondary antibody 1 h. Images were captured by a confocal laser scanning microscope (Fluoview FV1000, Olympus). Immunohistochemical staining was performed as described previously [28].

### Xenograft assays

The animal protocol was reviewed and approved by the animal ethics committee of China Pharmaceutical University. Nude mice were exposed to 3 Gy of X-rays for 24 h prior to the experiment. RPMI 8226 cells (2 × 10^7^ in 100μl PBS) were subcutaneously injected to the left flank of mice using a 25-gauge needle and a calibrated push button-controlled dispensing device (Hamilton Syringe). To prevent leakage, a cotton swab was held over the site of injection for 1 min. One week post transplantation, tumor diameters were measured using a Vernier caliper. The mice were randomly divided into three groups (n = 5 each): control, wogonin (40mg/kg) and wogonin (80mg/kg). Saline or wogonin was administrated via tail vein injection twice per week for 3 consecutive weeks. Tumor volumes were measured once every three days using the formula V = 0.5236 × d_1_^2^ × d_2_, where d_1_ and d_2_ is the shortest and longest diameter, respectively. The mice were killed 21 days after drug treatment.

### Isolation of MM cells from patients

We obtained multiple myeloma cells from patients (2 females and 3 males with ages between 25∼65 years old) without prior therapy (Zhongda Hospital of Southeast University, Nanjing, China). All experiments with human materials were performed upon approval of the appropriate ethics committees. Primary MM cells were collected by Ficoll-Paque (Pharmacia Biotech) as previously described [3].

### Statistical analysis

The data represent mean ± SEM from three independent experiments, except specifically indicated. Statistical analysis was performed using the two-paired Student's t-test or one-way ANOVA.

## SUPPLEMENTARY FIGURES AND TABLES


