# Supplementary material for: Wogonin inhibits multiple myeloma-stimulated angiogenesis via c-Myc/VHL/HIF-1α signaling axis
Source: Oncotarget. 2015 Dec 30;7(5):5715–27. doi: 10.18632/oncotarget.6796 (PMC4868716; doi:10.18632/oncotarget.6796)
Supplement: Supplementary file 1 [file oncotarget-07-5715-s001.pdf]

SUPPLEMENTARY FIGURES AND TABLES

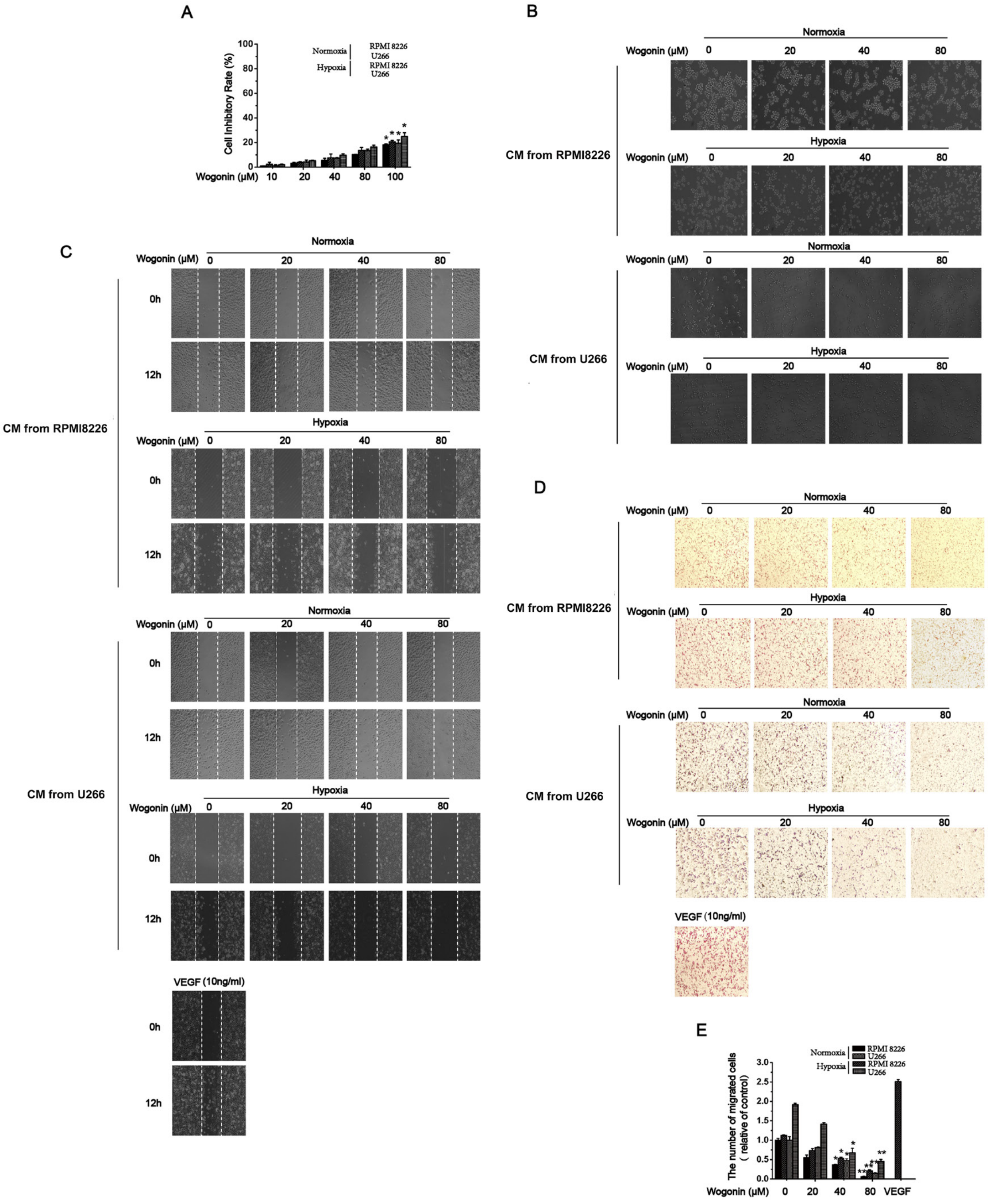

Supplementary Figure S1: Effects of Wogonin on MM-Stimulated Endothelial Cell Migration. A. RPMI 8226 and U266 cells were treated with various concentrations of wogonin (10, 20, 40, 80 and 100 μM) under normoxia and hypoxia for 24 h. Cell viability was determined using MTT assay. Data were expressed as percentages of the control (100%) as means ± SEM. n = 3 of three independent experiments (*p < 0.05). **B.** Morphologic analysis of MM cells cultured under normoxia and hypoxia for 24 h (magnification, ×200). **C.** HUVECs cultured in regular medium were stretched and extensively washed with PBS to remove away floating cells, followed by stimulation by conditioned medium from control- or wogonin-treated MM cells for 12 h. Cell migration was monitored. **D.** HUVECs seeded in transwell chambers were stimulated by VEGF or conditioned medium from control- or wogonin-treated MM cells for 12 h. Migrated cells were stained by H.E. **E.** Quantification of migrated cells as in *D*. Data are shown as means ± SEM (n = 3). *p < 0.05, **p < 0.01, one-way ANOVA.

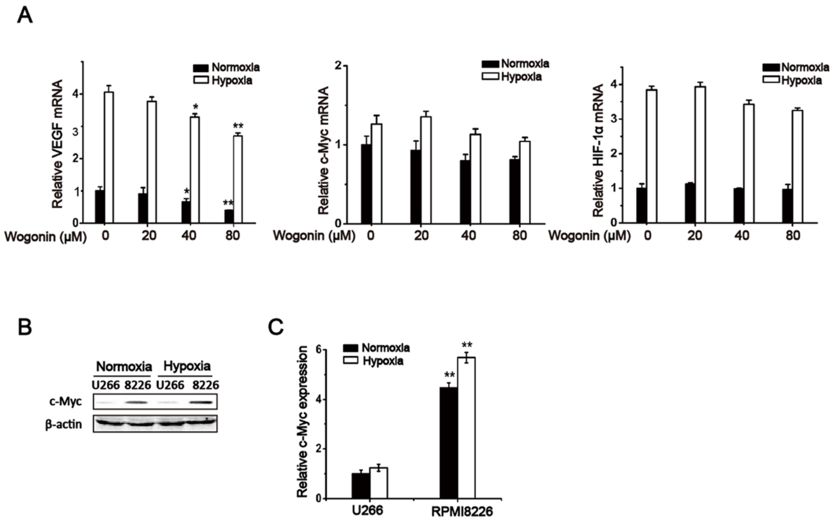

Supplementary Figure S2: Wogonin Affected VEGF but not c-Myc or HIF-1α mRNA Levels in MM Cells. A. RPMI 8226 cells were treated with wogonin at various concentrations (0, 20, 40 and 80 μM) for 24 h. Cell lysates were prepared and subjected to RT-qPCR analysis. **B.** RPMI 8226 cells and U266 cells were cultured under normoxic and hypoxic conditions and subjected to Western blot analysis using antibodies to c-Myc. **C.** Quantification of c-Myc protein level as in *B*. The results are representative of three independent experiments. Data are shown as means ± SEM (n = 3). *p < 0.05, **p < 0.01, one-way ANOVA.

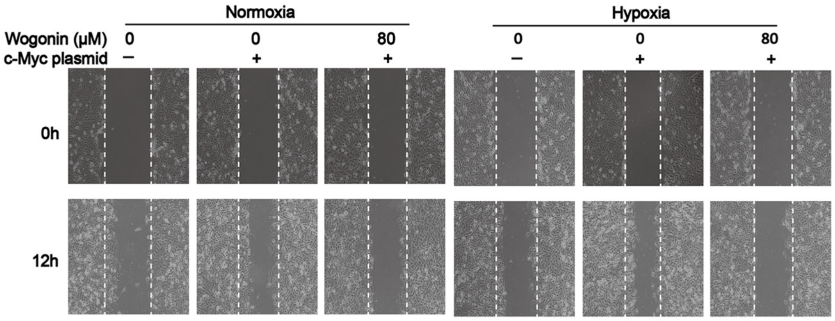

Supplementary Figure S3: Wogonin Inhibited c-Myc-Induced Endothelial Cell Migration. RPMI 8226 cells transfected with mock or c-Myc expressing vectors were cultured under normoxia and hypoxia for 24 h, followed by treatment with wogonin for an additional 24 h. Conditioned medium were harvested and applied to stimulate scratched HUVECs. Cell migration was then monitored.

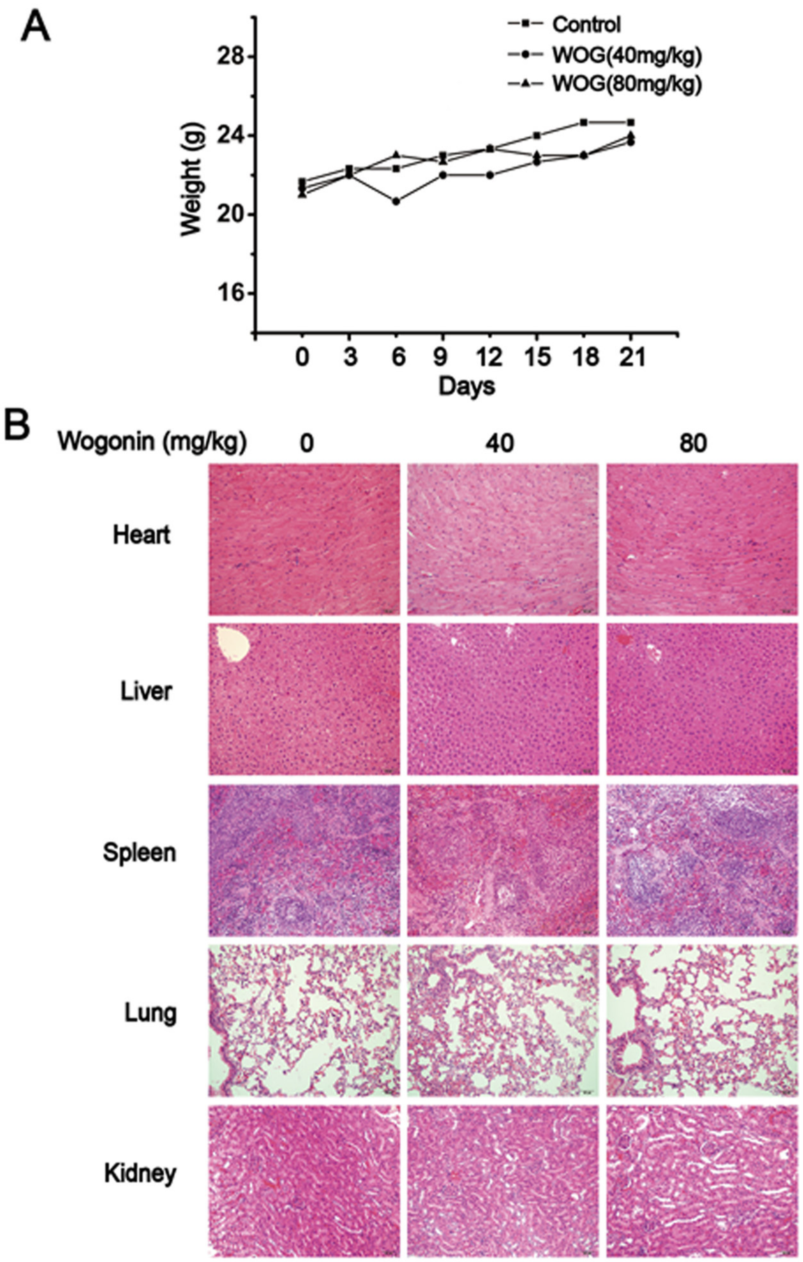

Supplementary Figure S4: Wogonin Did Not Affect Growth of Tumor-Bearing Mice without Inducing Histological Changes of Key Organs. A. 1×10^6 RPMI 8226 cells were subcutaneously (*s.c.*) injected in nude mice and the tumor bearing mice were randomly divided into 3 groups. Wogonin was administrated at doses of 0, 40, 80 mg/kg via intravenous (*i.v.*) injection every three days, and body weight of the mice were measured every three days. **B.** H&E staining of key organs derived from control- and wogonin-treated mice.

Supplementary Table S1: Hematological profiling of tumor-bearing nude mice

Hematological parameters	Control	Wogonin 40mg/kg	Wogonin 80mg/kg	Standard
White blood cells (×10^3 µl)	6.14/7.21	4.73/5.81	5.74/6.3	4.5/9.1
Platelet (×10^3 µl)	374/468	129/145	184/257	115/1037
Band neutrophils (%)	0.68/0.71	0.54/0.42	0.46/0.35	0-1
Lymphocytes (%)	49.1/50.2	53.8/61.7	52.7/54.9	49-82
Eosinophils (%)	0.24/0.18	0.04/0.07	0.04/0.08	0-3
Monocytes (%)	2.29/3.1	3.04/2.94	3.01/3.58	2-8
Mean corpuscular volume (fl)	43.4/45.6	47.2/43.1	45.9/48.2	41-60
Hematocrit(%)	49.8/46.1	44.2/41.7	48.9/40.3	34-50
Basophils (%)	0.14/0.23	0.24/0.36	0.34/0.51	0-3
Mean corpuscular hemoglobin (pg)	14.3/16.0	15.6/13.2	14.6/15.9	13-19
Mean corpuscular hemoglobin concentration (%)	32.9/34.7	33.0/33.1	31.7/34.2	30-39
Red blood cells (×10^3 µl)	11.47/9.37	9.36/9.33	10.65/11.29	7.51-16.1
Hemoglobin (g/dL)	16.1/15.9	14.6/13.7	15.5/14.8	12.8-16.1

Hematological profiling of tumor-bearing nude mice that are treated with PBS or wogonin (40 mg/kg and 80 mg/kg).Three mice per group were used. Standard ranges were obtained in-house from 100 normal BALB/c mice aged 8-12 weeks.

Supplementary Table S2: Primer sequences

Gene symbol	Organism	Forward primer (5′-3′)	Reverse primer (5′-3′)
c-Myc	Homo Sapiens	CGAGGAGGAGAACTTCTACCAGC	CGAGAAGCCGCTCCACATACAGTCC
HIF-1α	Homo Sapiens	GGCGCGAACGACAAGAAAAAG	CCTTATCAAGATGCGAACTCACA
VEGF	Homo Sapiens	CGCAGCTACTGCCATCCAAT	GTGAGGTTTGATCCGCATAATCT
VHL	Homo Sapiens	CCTTTGGCTCTTCAGAGATGC	GGTCTTTCTGCACATTTGGGTGG
GAPDH	Homo Sapiens	GGTGTGAACCATGAGAAGTATGACAAC	CCAGTAGAGGCAGGGATGATGTTC
